# Broadband picometer-scale resolution on-chip spectrometer with reconfigurable photonics

**DOI:** 10.1038/s41377-023-01195-2

**Published:** 2023-06-25

**Authors:** Chunhui Yao, Minjia Chen, Ting Yan, Liang Ming, Qixiang Cheng, Richard Penty

**Affiliations:** 1 Centre for Photonic Systems, Electrical Engineering Division, Department of Engineering, University of Cambridge, Cambridge, CB3 0FA UK; 2 GlitterinTech Limited, Xuzhou, 221000 China

**Keywords:** Imaging and sensing, Integrated optics

## Abstract

Miniaturization of optical spectrometers is important to enable spectroscopic analysis to play a role in in situ, or even in vitro and in vivo characterization systems. However, scaled-down spectrometers generally exhibit a strong trade-off between spectral resolution and operating bandwidth, and are often engineered to identify signature spectral peaks only for specific applications. In this paper, we propose and demonstrate a novel global sampling strategy with distributed filters for generating ultra-broadband pseudo-random spectral responses. The geometry of all-pass ring filters is tailored to ensure small self- and cross-correlation for effective information acquisition across the whole spectrum, which dramatically reduces the requirement on sampling channels. We employ the power of reconfigurable photonics in spectrum shaping by embedding the engineered distributed filters. Using a moderate mesh of MZIs, we create 256 diverse spectral responses on a single chip and demonstrate a resolution of 20 pm for single spectral lines and 30 pm for dual spectral lines over a broad bandwidth of 115 nm, to the best of our knowledge achieving a new record of bandwidth-to-resolution ratio. Rigorous simulations reveal that this design will readily be able to achieve single-picometer-scale resolution. We further show that the reconfigurable photonics provides an extra degree of programmability, enabling user-defined features on resolution, computation complexity, and relative error. The use of SiN integration platform enables the spectrometer to exhibit excellent thermal stability of ±2.0 °C, effectively tackling the challenge of temperature variations at picometer-scale resolutions.

## Abstract (web summary)

We propose an on-chip spectrometer design that leverages reconfigurable networks with distributed broadband filters, demonstrating a 30 pm resolution over a 115 nm bandwidth that can be readily scaled further.

## Graphical abstract

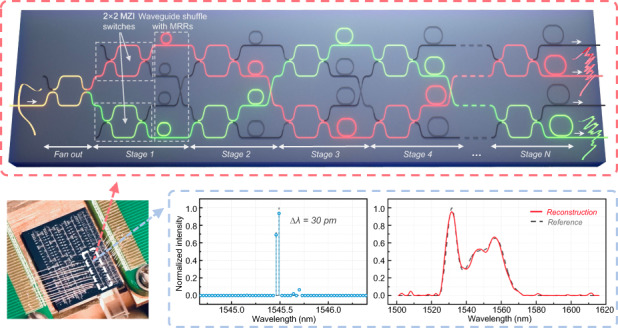

## Introduction

The optical spectrometer is one of the most essential tools in chemical and biological sensing, material characterization, and astronomical science^1,2^. Conventional benchtop spectrometers are usually built using bulky dispersive components, which makes them inadequate to meet the rapidly growing demands for compact and low-cost spectrum analysis, such as the wearable devices for healthcare monitoring^3,4^, smartphone or drone based remote sensing^5,6^, and space exploration^7^. Over the past decades, extensive efforts have been devoted from both academia and industry to develop miniaturized spectrometers. Nevertheless, the size reduction of spectrometers inevitably forces them to make trade-offs between resolution, bandwidth, signal-to-noise ratio, and etc.^8,9^. None of the scaled-down demonstrations so far can break the technical bottleneck to simultaneously achieve ultra-high resolution (down to picometer-scale) and broad bandwidth (>100 nm)^8,10^. These are, however, essential requirements towards an analytical spectroscopy tool for many biomedical sensing^11,12^ and industrial chemical monitoring applications^13,14^, and miniaturized optical imaging systems, for example, spectral-domain optical coherence tomography (SD-OCT) that demands both large imaging depth and high spatial resolution^15^.

**Fig. 1:**
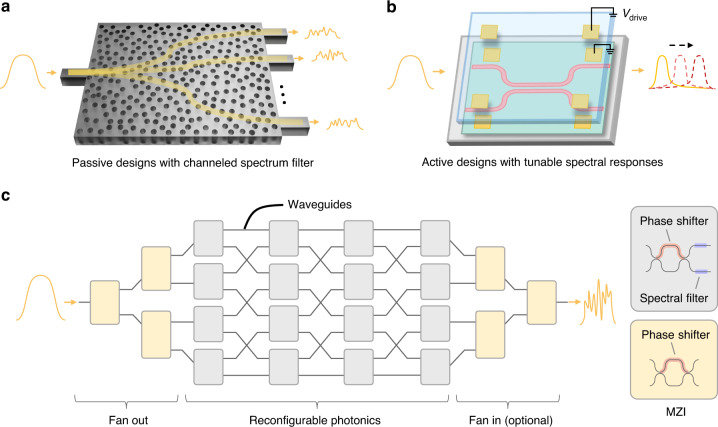
Concept of the proposed reconfigurable spectrometer. **a** Schematic of passive RS designs based on channeled spectrum filters with passive channel splitting. Here, we use a disordered scattering medium as an example. The reduction in the spectrum amplitude results from the inherent power splitting losses. **b** Schematic of active RS designs with tunable spectral responses using lumped structures, which inevitably have a high cross-correlation between sampling channels. Here, a MEMS-enabled tunable RS is shown as an example. **c** Concept illustration of our proposed reconfigurable RS. A reconfigurable network based on switchable elements is used to actively route the incident signal via different optical paths without inducing any splitting loss. Broadband spectral filters are distributed after each switchable element to generate a highly uncorrelated spectral response for each channel

Demultiplexing-and-detection spectrometers usually rely on dispersive elements or narrowband filters that decompose the incident light spectrally into either spatial or temporal detection channels^16,17^. This creates a linear mapping between the spectral components and channeled power. The channel number consequently defines the bandwidth-to-resolution ratio, which is bounded either by the minimum detectable power each channel or the acceptable device complexity/footprint. Leveraging compressive sampling, the reconstructive spectrometer (RS) emerges as a new paradigm for efficient spectrum acquisition. Using a limited number of sampling channels encoded with varied spectral responses, RSs can sample the entire incident spectrum with aggregated optical powers and resolve a larger number of spectral pixels, though trading off with more complex spectral-to-spatial mapping^18^. The nature of such underdetermined systems would best facilitate the development of on-chip spectrometers as minimum resources are required. However, RS would still require a large number of broadband sampling channels that are highly uncorrelated to approach an ultra-high bandwidth-to-resolution ratio. Figure 1a presents a generic schematic of reported miniaturized RSs based on passive spectrum filters, via, e.g., disordered scattering media^19–21^, metasurface-, photonic crystal-, or quantum dot-based filter arrays^22–24^. Whilst they represent the neatest form of an ultra-compact RS, the channel numbers can be restricted due to the passive splitting loss. Recent studies have also developed active RSs with tunable spectral responses, e.g., the detector-only RSs with tunable absorption spectra^25,26^, filter-based RSs with MEMS^27^ or thermally tunable resonators^28^, as illustrated by Fig. 1b. However, the ways of generating sampling channels reported so far by using lumped structures exhibit limited decorrelation, posing a constraint towards ultra-high bandwidth-to-resolution ratio, in accordance with the theory of compressive sensing^29^.

In this paper, we present a significant step towards an ultra-broadband picometer-scale resolution spectrometer on a photonic integrated chip by introducing a novel method with distributed filters for generating ultra-broadband pseudo-random spectral responses. The resulted sampling channels are thus highly uncorrelated, allowing computational reconstruction. We embed the distributed filters in a reconfigurable photonic network on a chip, as shown by Fig. 1c, in turn allowing a superior scalability in the number of sampling channels without sacrificing the decorrelation among them. By properly engineering the spectral properties of each distributed filter, the series of overlaid transmission spectra can form a sampling matrix with small self- and cross-correlation for effective information acquisition throughout the whole spectrum. Reconfigurable photonic circuits have found their applications in a variety of emerging fields including optical and quantum computing^30^, optical switching and signal processing^31^, and optical networking^32^, enabled by the silicon-on-insulator (SOI) platform^33^. Embedding filters in such a reconfigurable photonic network allows spectrum shaping. While the design can readily be implemented with the nanophotonic silicon circuits, we instead choose to use the CMOS-compatible silicon nitride (SiN) platform for its superior thermal robustness, as the temperature variations will limit the reconstruction accuracy when the spectrometer goes down to picometer-scale resolution^8^.

Micro-rings have been proposed to form a single-resonator spectrometer, leveraging its add-drop filtering to form a tunable local sampler^34^. In our design, a strategy to efficiently sample the entire input spectrum using a cascade of all-pass micro-ring resonators (MRRs) is employed. These all-pass ring filters operate in the over-coupling region with limited extinction ratios that minimizes the sampling loss. The capability of global sampling dramatically reduces the requirement on sampling times, i.e. temporal sampling channels. Using reconfigurable photonics, user-defined performance can be enabled providing an extra degree of programmability, depending on the trade-offs between resolution, computation complexity, and relative error. This can broaden its applications, covering use cases for both identifying signature spectral peaks with acceptable levels of performance^35^ and relative metrology with ultra-high resolution and low errors^36^. Using a moderate mesh of interconnected Mach-Zehnder interferometers (MZIs) that creates up to 256 reconfigurable states, we demonstrate an on-chip spectrometer with ultra-high resolution (<30 pm) and ultra-broad bandwidth (>115 nm), yielding, to the best of our knowledge, the highest bandwidth-to-resolution ratio of RSs reported to date (see detailed performance comparison in Supplementary Section S1). With experimentally fitted data and an equivalent level of measurement error, we further reveal that this approach can readily achieve single-picometer-scale resolution. Despite of the global sampling strategy that aggregates noise power at detection, we successfully resolve a narrowband laser signal with only 2 dB optical signal-to-noise ratio (OSNR). We further show that our device features excellent thermal stability of ±2.0 °C, thanks to the SiN platform, indicating a clear path towards on-chip spectrometers with an accuracy comparable to or even beyond benchtop spectrometer products.

## Results

### Principle and design

The operation principle of the RS can be mathematically described such that when an unknown incident spectrum $\varPhi \left(\lambda \right)$ propagates through one of its broadband sampling channels with a spectral response $S\left(\lambda \right)$, the output signal intensity is:

1 $$I=\int S(\lambda ){\Phi }(\lambda )d\lambda$$

**Fig. 2:**
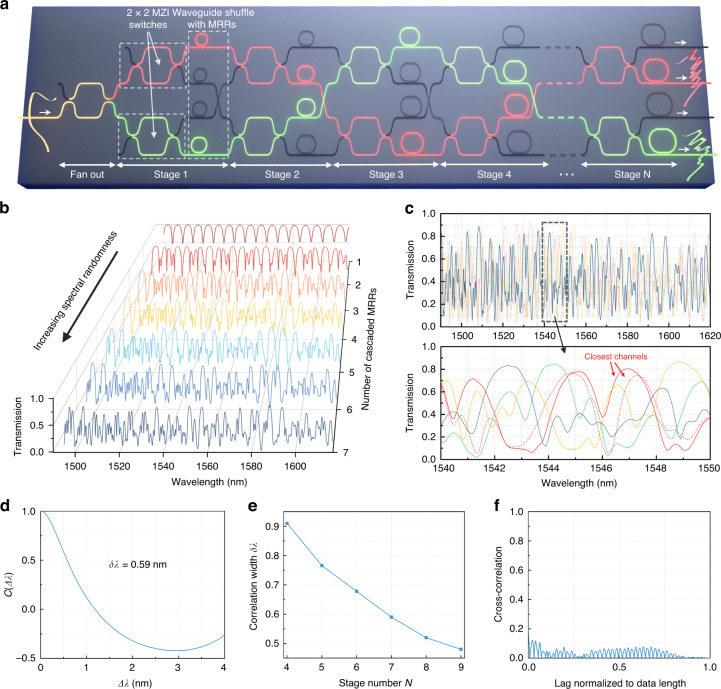
Reconfigurable spectrometer based on meshes of MZIs and distributed MRRs **a** Schematic of our demonstrated reconfigurable spectrometer based on meshes of switchable MZIs and distributed all-pass MRR filters. The green and red light paths represent two configured optical channels passing through different combinations of MRRs, thus having different spectral responses. **b** Simulated transmission spectra when different number of MRRs are cascaded, which results in the generation of pseudo-random spectral responses using distributed filters. **c** Several example channel spectral responses from a 7-stage design. The red solid and dashed lines represent the closest pair of channels that share six of the same MRRs but still feature a significant spectral difference. **d** The calculated spectral self-correlation function *C* (Δλ) when the stage number is 7, with a correlation width δλ of 0.59 nm. **e** The correlation width δλ at different stage numbers. **f** The absolute value of the averaged cross-correlation between one specific channel and all the other channels

Likewise, when the incident signal passes through *i* channels, each encoded with distinctive spectral responses, Eq. (1) can be discretized and written in matrix format:

2 $${I}_{i\times 1}={S}_{i\times j}{\varPhi }_{j\times 1}$$

where *j* defines the spectral pixels in the incident spectrum, i.e., defining the bandwidth-to-resolution ratio. With mathematical algorithms and engineering of the sampling matrix ${S}_{i\times j}$, Eq. (2) can be solved under the case when $i\ll j$, which reveals the key privilege of the global sampling strategy of RSs. Figure 2a shows the schematic of our demonstrated spectrometer with meshes of switchable MZIs and distributed MRR filters. Except for the fan-out, each stage contains two 2 × 2 balanced MZIs in vertical arrangement and are followed by a waveguide crossing that interconnects the upper and lower MZIs between adjacent stages, forming a chain-link fence pattern. Note that the switching of MZIs requires a π phase shift, and thus has no requirement for fine tuning, being resilient to possible electrical fluctuations^27,28^. A set of MRRs is allocated to the four MZI outputs at each stage and act as distributed broadband spectral filters. Therefore, by reconfiguring the state of each MZI (i.e., to either cross or bar state), an incident light can be successively routed through different paths to establish varying spectral responses. As each MZI cell is set in a binary fashion, the channel number grows exponentially with the increase in number of stages. Numerically, the stage number *N*, the number of MZI ${N}_{{MZI}}$, and the number of total channels ${N}_{{channel}}$ follow:

3 $$\left\{\begin{array}{c}{N}_{{channel}}={2}^{N+1}\\ {N}_{{MZI}}=2N+1\end{array}\right.$$

While a great number of sampling channels can be created following this topology, a successful RS still lies in tailoring the channel spectral responses (i.e. the sampling matrix). In general, each channel’s transmission spectrum should have a small self-correlation length, namely rapid fluctuations in the wavelength domain to allow high sampling resolution. In addition, the transmission spectra of different channels should be disparate (ideally, orthogonal) to provide uncorrelated spectrum sampling. To efficiently achieve both requirements, we develop a distributed spectral filtering method with all-pass racetrack MRRs. By tweaking the geometries of an MRR, its spectral response, including the free spectral range (FSR), full width at half maximum (FWHM), extinction ratio (ER), and resonance wavelengths, can be fully manipulated. Since each reconfigured path comprises a unique combination of MRRs, the overlaid transmission spectrum can exhibit strong diversities among different channels. Note that we design the MRRs to be over-coupled with small extinction ratios, aiming to develop a high global sampling efficiency and induce a low sampling loss. Detailed discussions about the parameter design can be found in Methods and Supplementary Section S2. Figure 2b shows the evolution of the transmission spectrum as the number of cascaded MRRs increases. As can be seen, the overlaid spectra quickly lose the original periodicities and presents increasingly randomness. Figure 2c plots the transmission spectra of representative paths in a 7-stage design, including two channels with six identical MRRs in their path. Note that such a case only happens twice for any channel (see Supplementary Section S3 for more details), suggesting sufficient overall diversity in the channel spectral responses.

To further quantify the performance of the spectral response generated by the distributed MRRs, the spectral self-correlation of each channel can be assessed by:

4 $$C\left(\triangle \lambda ,i\right)=\frac{\left\langle I(\lambda ,i)I(\lambda +\triangle \lambda ,i)\right\rangle }{\left\langle I(\lambda ,i)\right\rangle \left\langle I(\lambda +\triangle \lambda ,i)\right\rangle }-1$$

where $I(\lambda ,i)$ is the transmission intensity at channel *i* for wavelength *λ*, and $\left\langle \cdots \right\rangle$ corresponds to the average over wavelength. $C\left(\triangle \lambda ,i\right)$ can then be averaged over all the channels, as $C\left(\triangle \lambda \right)$. The half width at half maximum (HWHM) of $C\left(\triangle \lambda \right)$, namely the spectral correlation width δλ, can be used to denote the channel sampling resolution^37^. Figure 2d presents the calculated $C\left(\triangle \lambda \right)$ for a 7-stage case with a δλ of 0.59 nm. It should be noted that the δλ can be further reduced by increasing the number of cascaded MRRs, as illustrated by Fig. 2e. Also note that the channel sampling resolution refers only to the overall sampling efficiency of individual filters^38^. In the following section, we show that the resolved resolution can significantly exceed the channel sampling resolution if a sufficient number of channels is used. On the other hand, the cross-correlations between the channels are calculated to be mostly below 0.1, indicating that the spectral response of different channels are highly uncorrelated, as shown by Fig. 2f.

### Experimental characterization

**Fig. 3:**
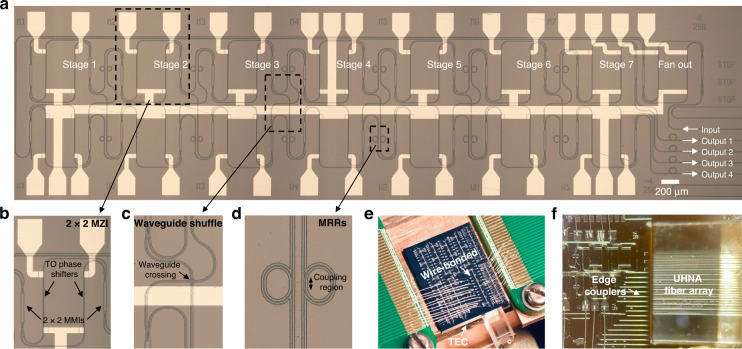
Fabricated spectrometer and its packaging. **a** Microscope image of the fabricated on-chip spectrometer in a 7-stage design. **b**–**d** Enlarged views of a 2 × 2 thermo-optic MZI, interconnected waveguide shuffle, and a pair of MRRs, respectively. **e** Photograph of the photonic chip wire bonded to a customized PCB board with a thermoelectric cooler (TEC) placed underneath. **f** The coupling facet between the edge couplers and the UHNA fiber array

Figure 3a shows the fabricated reconfigurable spectrometer device and its packaging. We choose a 7-stage design in this demonstration, out of consideration of device performance, footprint, and system complexity. Figure 3b–d shows the enlarged views of the key building blocks, respectively, including a 2 × 2 thermo-optic (TO) MZI with multimode interferometers (MMIs), interconnected waveguide shuffle, and a pair of MRRs. The chip is wire-bonded to a customized PCB board for electrical fan-out, as shown in Fig. 3e. Ultra-high-NA (UHNA) fiber array is used for optical coupling shown by Fig. 3f.

**Fig. 4:**
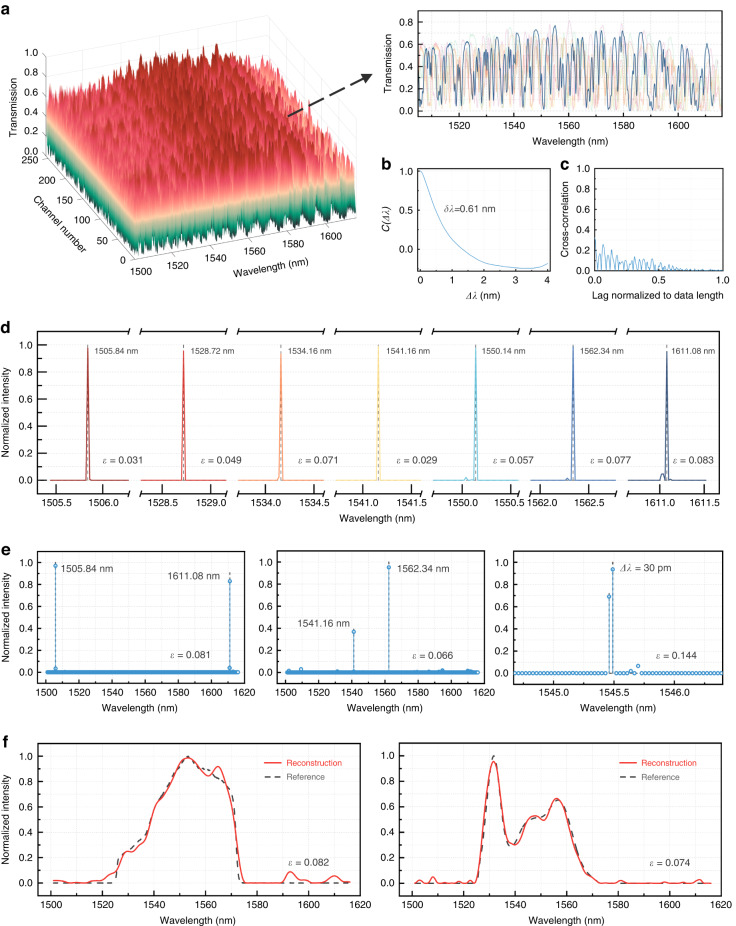
Calibration and testing of the reconfigurable spectrometer. **a** Measured spectral responses for the 256 reconfigured sampling channels. The inset shows the transmission spectra of several examples of the sampling channels. **b**, **c** The calculated spectral self-correlation function (Δλ) and the averaged cross-correlation between 256 channels, respectively. **d** Reconstructed spectra for a series of narrowband spectral lines across a 115 nm bandwidth. The black dashed lines mark their center wavelengths. **e** Reconstructed spectra for dual spectral lines at different wavelengths: dual peaks at 1505.84 nm and 1611.08 nm (left), dual peaks at 1541.16 nm and 1562.34 nm (middle), and dual peaks with a spectral spacing of 30 pm, respectively. **f** Reconstructed spectra for continuous, broadband signals: the ASE from an SOA (left) and an EDFA (right) filtered by a bandpass filter, respectively

We first calibrate the whole chip and derive the look-up table detailing the binary state bias voltages of all 7-stage MZIs. We then calibrate the channel spectral responses by launching the amplified spontaneous emission (ASE) of a semiconductor optical amplifier (SOA) through the chip and measuring the transmission spectra with a commercial spectrum analyzer (see Supplementary Fig. S4) for each channel. Figure 4a shows the spectral responses of the 256 sampling channels. The inset depicts the transmission spectra of several representative sampling channels, showing a high degree of randomness in the wavelength domain with a large fluctuation range. Specifically, the δλ is calculated to be 0.61 nm, aligning well with the simulation results, and the cross-correlation is mostly below 0.2, as shown by Fig. 4b and c, respectively.

Subsequently, we launch light from distributed feedback laser (DFB) diodes at different wavelengths as narrowband signal inputs to characterize the device. An electrical control plane programmed via a microcontroller unit is developed to automatically deploy the bias voltages for all sampling channels and collect the output signal intensity $I$ from an optical power meter temporally, which completes the whole measurement within 0.3 s (see more details in Supplementary Section 4). Here, the sampling spectral window is set between 1501 nm and 1616 nm. Accordingly, the sampling matrix ${S}$ is extracted from the pre-calibrated channel spectral responses. To solve Eq. (2) under the case of $i\ll j$, nonlinear optimization algorithms can be applied to find the incident spectrum that can minimize:

5 $${{||I}-S\varPhi {||}}_{2}{\rm{subject\; to}}\,0\le \varPhi \le 1$$

We use the CVX optimization algorithm for spectrum recovery^39^. The relative error is used to quantify the reconstruction accuracy as^38^:

6 $$\varepsilon =\frac{{{||}{S}_{0}-{S||}}_{2}}{{{||}{S}_{0}{||}}_{2}}$$

where *S* is the reconstructed spectrum, ${S}_{0}$ is reference. Figure 4d shows the resolved spectra for all the laser signals with calculated error $\varepsilon$ ranging between 0.029 to 0.083, indicating high reconstruction accuracy. The FWHM of the resolved peaks maintain consistently at about 20 pm. We also test the case of dual spectral lines by simultaneously launching two laser signals at different wavelengths. The spacing between the two spectral lines is reduced from 105 nm down to 30 pm, as shown by Fig. 4e. It can be seen that all peak intensities can be well distinguished, illustrating an ultra-high resolution of 30 pm.

Besides, we demonstrate the reconstruction of continuous broadband spectra, which is widely recognized as a more challenging case for RS. This is because the contrast of spectral patterns in the sampling filters gets compromised during the integration of light over wavelength^40^. This is particularly pronounced when the sampling matrix is ill-conditioned that minor measurement error or noise may lead to considerable reconstruction error. Hence, we introduce additional regulation terms to Eq. (5) to improve the reconstruction accuracy^41^, as

7 $${{||I}-S\varPhi {||}}_{2}+{\alpha {||}{\Gamma }_{1}\varPhi {||}}_{1}+{\beta {\rm{||}}{\Gamma }_{2}\varPhi {\rm{||}}}_{2}{\rm{subject\; to}}\,0\le \varPhi \le 1$$

where the *α* and *β* are two weight coefficients that can be optimized via cross-validation analysis^27^, while Γ_1_ and Γ_2_ correspond to the matrices used to compute the first and second order derivatives of $\varPhi$, respectively. The experimental procedures are the same as those for the narrowband signals expect that the light sources under test are the ASE spectra of an SOA and an Erbium-doped fiber amplifier (EDFA) followed by a bandpass filter, respectively. Figure 4f shows the reconstructed broadband spectra with $\varepsilon$ of 0.082 and 0.074, respectively. We attribute the reconstruction error to the calibration variation owning to the use of an ASE source that is unpolarized. This can, however, be effectively solved by using an ultra-broadband tunable laser source instead. In addition, advance reconstruction algorithms with better noise tolerance may also help provide higher reconstruction accuracy^29^, but are outside of the scope of this paper.

**Fig. 5:**
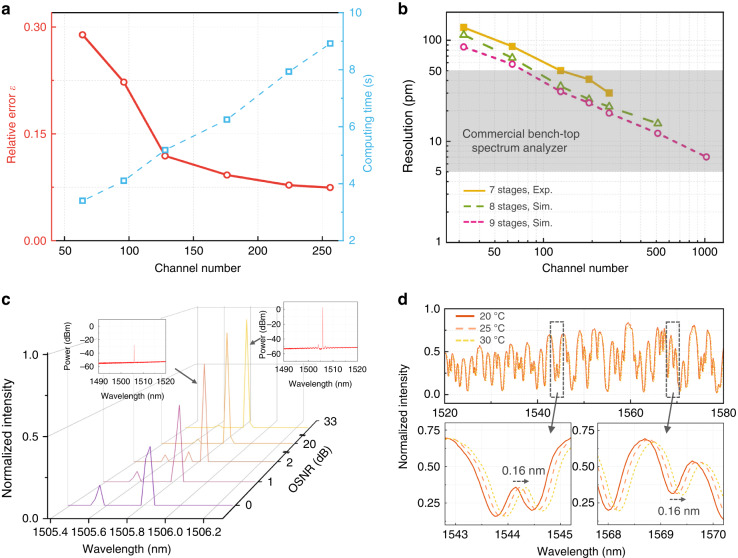
Further explorations about the programmability, resolution scalability, sensitivity, and thermal stability of the reconfigurable spectrometer. **a** Reconstruction accuracy and computing time vs. the channel number. **b** Spectrometer resolution vs. the channel number, including the experimental results from a 7-stage design and the simulated results from 8- and 9-stage designs. The continuous downward trends reveal the path towards picometer-scale spectral resolution, reaching a same level with commercial bench-top products (YOKOGAWA AQ series47). **c** The reconstructed spectra of a narrowband signal under different OSNR with aggregated noise. The insets show two spectra of inputs with constant noise floor at −55 dBm. **d** The measured spectral response of one sampling channel at different temperatures. The insets show the small redshift of the spectral response with increasing temperature

Thanks to the global sampling strategy, only dozens of sampling channels would be capable of reconstructing the input spectrum coarsely. This offers great flexibility in programming the spectrometer by grouping different combinations of sampling channels that can decide the trade-offs between its resolution, computational complexity, and reconstruction accuracy. This facilitates a user-definable performance to suit different application scenarios^8^. To reveal the underlying links, we examine the relative error against channel number, along with the consumed computing time (running the MATLAB CVX algorithm on a Xeon 7980 CPU), utilizing the ASE spectrum from an EDFA as an example case. As depicted in Fig. 5a, the reconstruction error steadily drops with an increase in sampling channels, but at the cost of a linearly increasing computation time. Furthermore, Fig. 5b illustrates the correlation between reconstruction resolution (validated by solving dual spectral lines) and the channel number. With experimentally fitted data and an equivalent level of measurement error, we also perform simulations with larger meshes of discrete spectral filters, i.e., 8 and 9 stages. It can be seen that the resolution follows an evident downward trend as the channel number increases, approaching a spectral resolution down to single picometer scale.

Another unique feature offered by the design of active fan-out and routing is high sensitivity. We investigate this feature using a “convergence test” by recovering a decreasing power of a single line spectrum generated by a DFB laser combined with a noise floor maintained at about −55 dBm. This case, of a narrowband signal with broadband noise, represents the worst-case OSNR since the RS captures aggregated power per channel. We managed to reconstruct the narrowband signals with a peak power down to −27.5 dBm, i.e., an OSNR with aggregated noise of only 2 dB, as illustrated by Fig. 5c. Further degradation of OSNR leads to distortion of reconstructed spectral shape with non-negligible error in wavelengths. A tunable bandpass filter can be implemented to work in tandem with the reconfigurable spectrometer to improve the sensitivity by removing excess noise outside of the signal band^42,43^.

Temperature variation is also an issue for spectrometer performance as it may distort the channel spectral responses^28^. Owning to the low thermo-optic efficiency of SiN, the channel spectral response of our device only redshifts by about 0.16 nm with a 10 °C temperature rise, while the waveform remains unchanged, as shown by Fig. 5d. Accordingly, we model the thermal stability of the spectrometer, as detailed in Supplementary Section S5. The results indicate that up to a temperature change of ±2.0 °C, the input spectra can still be accurately recovered. In practice, on-chip temperature stabilization techniques can be used to minimize temperature drift^44^. In addition, it is feasible to offset the spectral response shift from the algorithmic perspective via real-time temperature monitoring.

## Discussion

In this paper, we introduce a novel global sampling method with ultra-broadband distributed filters that creates highly uncorrelated sampling channels. By populating the discrete filters in a reconfigurable photonic circuit, we demonstrate a broadband picometer-scale resolution spectrometer on a single photonic chip. The use of a global sampling strategy significantly reduces the need of sampling channels as to reconstruct an ultra-large number of spectral pixels, which in turn produces programmability that offers user-defined features, broadening its applications. Based on a commercial SiN photonic integration platform, we realize an on-chip spectrometer using a 256-state photonic network using embedded all-pass MRRs that demonstrates a 30 pm spectral resolution over a 115 nm bandwidth. This, to the best of our knowledge, represents the highest bandwidth-to-resolution ratio of RSs ever reported. We also show that the current level of performance in the fabricated device can be further improved by scaling up the embedded reconfigurable network to approach a picometer-scale spectral resolution. Furthermore, using basic building blocks with ultra-broadband responses, e.g., MMIs and waveguide crossings^45,46^, the recovered spectral range can be extended to a few hundreds of nanometers (please find more simulation results in Supplementary Section 1). Thereby, we foresee that this design would lead to a new class of chip-scale spectrometer with comparable performance levels to commercial benchtop spectrum analyziers^47^.

The use of SiN, a CMOS-compatible integration platform, permits the reuse of processes that have been developed to build complex photonic integrated circuits^48^. This means a route to mass-manufacturing at low-cost. Furthermore, the ultra-low thermal sensitivity of SiN brings an additional benefit of temperature robustness, which is critical in scenarios, such as in vivo health monitoring^49^. The sampling speed can be escalated by upgrading the electrical control plane for a higher data transmission rate. To trade off for smaller footprint, higher reconfiguration speed, or better electro-optic efficiency, other integration platforms, such as silicon, indium phosphide, lithium niobate, or heterogeneous integration schemes^50,51^, can also be exploited. The demonstrated chip-scale reconfigurable spectrometer can play a role in a vast range of applications, from the biomedical sensing for blood glucose or urea, to the industrial chemical detection for fuel or water pollution^18^. More importantly, the proposed spectrometer can be seamlessly integrated into optical imaging systems for real-time, noninvasive detections, such as OCT—a standard imaging technique for ophthalmologic care. The application of chip-scale high-performance spectrometers holds a great promise to reduce the size and cost of OCT systems for diagnostic applications^36^.

## Methods

### Distributed spectral filters design with MRRs

In pursuance of small self-correlation length and large diversity of the spectral responses, we conclude a few key design criteria to the MRR-based filters.

1. Small FSR is desired to induce rapid fluctuations for high sampling resolution. Meanwhile, the FSR of each set of MRRs must differ to break the periodicity of the overlaid spectra. In this design, the FSR of the MRRs varies from 4.7 nm to 8.1 nm.
2. Small finesse (i.e. large FWHM) is required to perturb the spectrum efficiently, and we choose to use over-coupled MRRs and set the finesse between about 5 to 7.
3. Modest ER is favored in such a cascading scheme and thus the intensity contrast in the overlaid spectra can be maximized without excess loss. Here, the MRRs are tailored to allow the transmission intensity to vary in between 0 and 0.9.
4. The resonance peaks of each set of MRRs are located at different wavelengths to ensure the diversity among paths. This is achieved by adjusting the circumferences of MRRs.

### Chip fabrication, design, and loss analysis

The chip was fabricated via the CORNERSTONE SiN multi-project wafer run using standard DUV lithography with a feature size of 250 nm. The integration platform includes a 300 nm thick LPCVD SiN layer, a 3 µm buried oxide layer, and a 2 µm silicon dioxide top cladding. The size of the fabricated device is about 2.0 × 7.6 mm^2^. The single-mode waveguides in the circuit are fully etched strip waveguide with a width of 1200 nm, featuring a propagation loss of <1 dB cm^−1^. We design and optimize the basic building blocks, including 2 × 2 MMI, waveguide crossing, and edge coupler, using a commercial simulator, i.e., Lumerical FDTD Solution. The cascaded 2 × 2 MMI couplers are believed to impose the bandwidth limitation of the spectrometer and it is designed to have a 120 nm bandwidth with an insertion loss of <0.5 dB. Each 2 × 2 balanced MZI cell is equipped with two thermal tuners that get separated by over 300 µm to suppress thermal crosstalk. The extinction ratio of MZI cells is characterized to be over 30 dB. The edge couplers are designed to have a mode diameter of about 3.5 µm in a reverse taper structure. Thus, an UHNA fiber array is applied with a matched mode size, and this yields a coupling loss of about 2.8 dB per facet. A reconfigurable photonic network chip without the distributed MRR filters is fabricated on the same die for loss analysis and normalization. The measured on-chip loss is about 4.9 dB at the wavelength of 1550 nm. This can be mainly attributed to the excess loss of MMIs and the propagation loss of waveguides.

### Electrical and thermal control

The electrical control signals are initially generated from a microcontroller unit (MCU), which is programmed to transmit the pre-calibrated voltage look-up table to a high-resolution digital-to-analogue converter (DAC) at a transmission rate of 750 kb s^−1^. Accordingly, the DAC outputs the analog electrical signals that are subsequently amplified by a customized driving board and imported into the spectrometer chip for automatic channel sweeping. An analogue-to digital converter (ADC) module embedded in the MCU is used to collect the real-time output signals from the photodiode. A thermoelectric cooler is placed underneath the chip for global temperature stabilizing, which is set to be 25 °C during our measurements.

## Supplementary information

Supplementary material of the paper

## Data Availability

Data underlying the results presented in this paper are available from the authors upon reasonable request.
