# Supplementary material for: Broadband picometer-scale resolution on-chip spectrometer with reconfigurable photonics
Source: Light Sci Appl. 2023 Jun 25;12:156. doi: 10.1038/s41377-023-01195-2 (PMC10290986; doi:10.1038/s41377-023-01195-2)
Supplement: Supplementary file 1 — Supplementary material of the paper [file 41377_2023_1195_MOESM1_ESM.docx]

**Supplemental Information**

**Broadband picometer-scale resolution on-chip spectrometer with reconfigurable photonics**

Chunhui Yao^1^, Minjia Chen^1^, Ting Yan^2^, Liang Ming^2^, Qixiang Cheng^1,2*^, Richard Penty^1^

1. Centre for Photonic Systems, Electrical Engineering Division, Department of Engineering, University of Cambridge, Cambridge, CB3 0FA, UK

2. GlitterinTech Limited, Xuzhou, 221000, China

*E-mail : qc223@cam.ac.uk

1. **Performance comparison with the state-of-the-art miniatured spectrometers**

In Fig. S1, we summarize the resolution and bandwidth, the two most important metrics, of state-of-the-art miniatured spectrometers based on dispersive optics, narrowband filtering, Fourier transform and computational reconstruction, respectively^S1–S25^. It can be seen that these demonstrations have clear trade-offs between the resolution and bandwidth, with bandwidth-to-resolution ratios mostly limited to the order of dozens or hundreds. In comparison, the proposed reconfigurable spectrometer effectively breaks such a limitation by the use of a global sampling strategy, decoupling the trade-off between resolution and bandwidth. By further scaling up the reconfigurable network, single-picometer-scale resolution can be readily achieved, revealed by rigorous simulations (see Fig. 5(b)). The operation bandwidth can also be improved by applying dispersion engineering techniques on the individual photonic building blocks. For example, in ref^S26^, the authors experimentally demonstrated a 2×2 MMI coupler with an over 300 nm bandwidth using a sub-wavelength grating (SWG) structure. As the bandwidth of our fabricated spectrometer is primarily limited by the conventional MMI, which has a bandwidth of about 120 nm, employing the SWG-based broadband MMIs is expected to extend the range of pseudo-random spectral responses to 300 nm. Accordingly, we simulated the spectrometer performance using a 9-stage reconfigurable network with SWG-based MMIs and achieves an ultra-high resolution of about 8 pm over the 300 nm bandwidth. This further illustrates that our design scheme can efficiently overcome the bandwidth-resolution trade-off without introducing additional on-chip resource consumption.


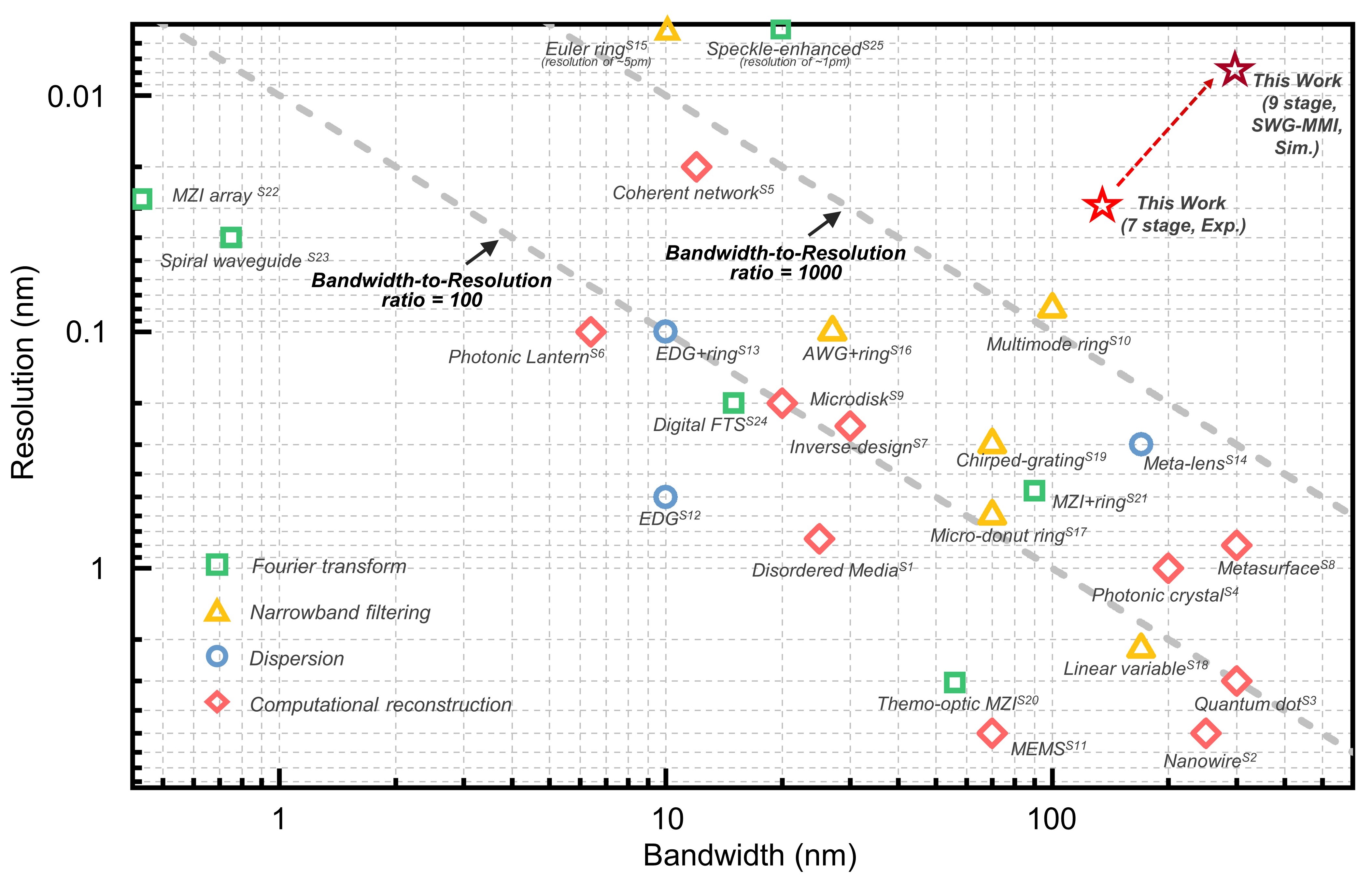


**Figure S1 | Resolution and bandwidth of the state-of-the-art miniaturized spectrometers.** Comparison with various miniatured spectrometers regarding the resolution and bandwidth. The dashed lines represent the bandwidth-to-resolution ratios at different orders of magnitude, clearly showing the performance breakthrough accomplished by our reconfigurable spectrometer.

Furthermore, we also highlight other important merits that comparisons are made in terms of footprint, sampling time, thermal stability, and control complexity, with representative spectrometer designs, as listed in Table S1. We attribute the large footprint of our demonstration to the use of SiN platform that has a smaller index contrast than the nanophotonic silicon platform. By implementing the same device on a standard 220 nm SOI platform, the footprint can be readily reduced to about 0.7 mm^2 S27^. However, as a trade-off to the footprint, our device undoubtedly shows the best thermal robustness, which is a vital thread to spectrometers working at picometer-scale resolutions.

Table S1. Merit comparison with several typical spectrometers

| **Ref** | **Scheme** | **Resolution (nm)** | **Bandwidth (nm)** | **Footprint (mm^2^)** | **Sampling time** | **Thermal stability** | **Control complexity** | **CMOS compatible** |
| --- | --- | --- | --- | --- | --- | --- | --- | --- |
| S1 | Disorder media | 0.75 | 25 | 1.25*10^-3^ | One-shot | ±4°C | N.A. | Yes |
| S2 | Nanowire | 5 | 250 | 0.1 mm | One-shot | N.M. | N.A. | No |
| S8 | Quantum dot | 2-3 | 300 | 100 | One-shot | N.M. | N.A. | No |
| S9 | Micro disk | 0.2 | 20 | 0.04 | N.M. | ~±0.12°C | High | Yes |
| S10 | Multimode ring | 0.08 | 100 | 0.7*10^-3^ | 375 s | ±0.014°C | High | Yes |
| S16 | AWG + ring | 0.1 | 27 | 9 | N.M. | ~±0.67°C | High | Yes |
| S19 | Chirped grating | 0.3 | 70 | 448 | One-shot | N.M. | N.A. | Yes |
| S25 | Spatial FT | 0.0011 | 6.2 | 13.9 | One-shot | N.M. | N.A. | Yes |
| This work | Reconfigurable photonics | 0.03 | 115 | 15.2 | <0.3 s | ±2°C | Low | Yes |

*N.A.: Not applicable; N.M.: Not mentioned.

**2. Geometric design of MRRs**

Following the design criteria discussed in Methods, this section further elaborates the geometric design of all-pass MRRs. Figure S2a shows the schematic of a racetrack ring resonator, where *R*, $L_{c}$, *G*, and *W* denote the ring radius, coupling length, gap distance, and waveguide width, respectively. The transmission of a racetrack MRR can be described as^S28,S29^:

$T=\frac{\alpha^{2}+r^{2}-2\alpha rcos\left( \theta\right)}{{1+\alpha}^{2}r^{2}-2\alpha rcos\left( \theta\right)}$ (S1)

where $r$ is the self-coupling coefficient, $\alpha$ is the loss coefficient, and $\theta$ is the single-pass phase shift of the ring. Based on Eq. S1, the FSR, FWHM, resonance wavelength $\lambda_{res}$, and transmission intensity at resonance wavelength $T_{res}$ can be derived as:

$FSR=\frac{\lambda^{2}}{n_{g}L}$ (S2)

$FWHM=\frac{(1-r\alpha)\lambda^{2}}{\pi n_{g}L\sqrt{r\alpha}}$ (S3)

$\lambda_{res}=\frac{n_{eff}L}{m}, m=1, 2, 3\cdots$ (S4)

$T_{res}=\frac{{(r-\alpha)}^{2}}{{(1-r\alpha)}^{2}}$ (S5)

where *L* is the circumference of the ring, $n_{eff}$and $n_{g}$ are the effective and group index of the waveguide, respectively. The ratio between FSR and FWHM is known as the finesse, suggesting the sharpness of resonances relative to their spacing. Figure S2b-c show the calculated maps of $T_{res}$ and the reciprocal of finesse respectively, for different combinations of $\alpha$ and $r$. As in a cascading scheme, the $T_{res}$ of each MRR should be set in a proper range so that the intensity contrast in the overlaid transmission spectra can be maximized (i.e., to maximize the fluctuations in intensity) without causing excessive loss. For a 7-stage design, a $T_{res}$ between 0.4 to 0.5 is preferred to allow the overlaid transmission intensity to vary in between 0 and 0.9. Meanwhile, each resonance peak shall have a large FWHM to efficiently perturb the spectrum, which requires a relatively small finesse. Another design consideration is that the resonance wavelengths for each set of four MRRs should locate differently in the wavelength domain to ensure large diversity, so that the FSR should be at-least four times larger than the FWHM to avoid overlapping between the resonance peaks. Hence, we choose to use over-coupled MRRs and tailor the finesse to be around 5 to 7. Accordingly, the insets in Fig S2b-c present the optimal range of $\alpha$ and $r$ for the MRRs.

In our design, the gap *G* is fixed as 300 nm considering the minimal feature size, and the waveguide width *W* is set to be 1.2 µm for single-mode operation. Hence, the $\alpha$ and $r$ are determined by the radius *R* and coupling length $L_{c}$. FDTD simulations show that the target range of $\alpha$ and $r$ can be achieved when the *R* and $L_{c}$ are between about 27 µm to 46 µm, and 5 µm to 14 µm, respectively. Correspondingly, the circumference of the racetrack can vary between 183 µm and 317 µm, enabling a small FSR ranging between 4.7 nm to 8.1 nm ($n_{g}=1.62$ at 1550 nm). In order to break the periodicity of the overlaid spectra, the FSR of different sets of MRRs is designed to gradually increase from 4.7 nm to 8.1 nm, i.e., with increasing radius *R* and coupling length $L_{c}$. In addition, the resonance wavelengths of each set of MRRs are carefully optimized to ensure that they are separated in the wavelength domain, creating distinctive spectral filtering. This can be easily achieved by slightly adjusting their circumferences (see Eq. S4). Following the above design criteria, Fig. S2d-f shows the transmission spectra of different sets of MRRs in a 7-stage design (taking stage 2, 4 and 6 as examples), showing the varying FSR and the resonance peaks at different wavelengths.


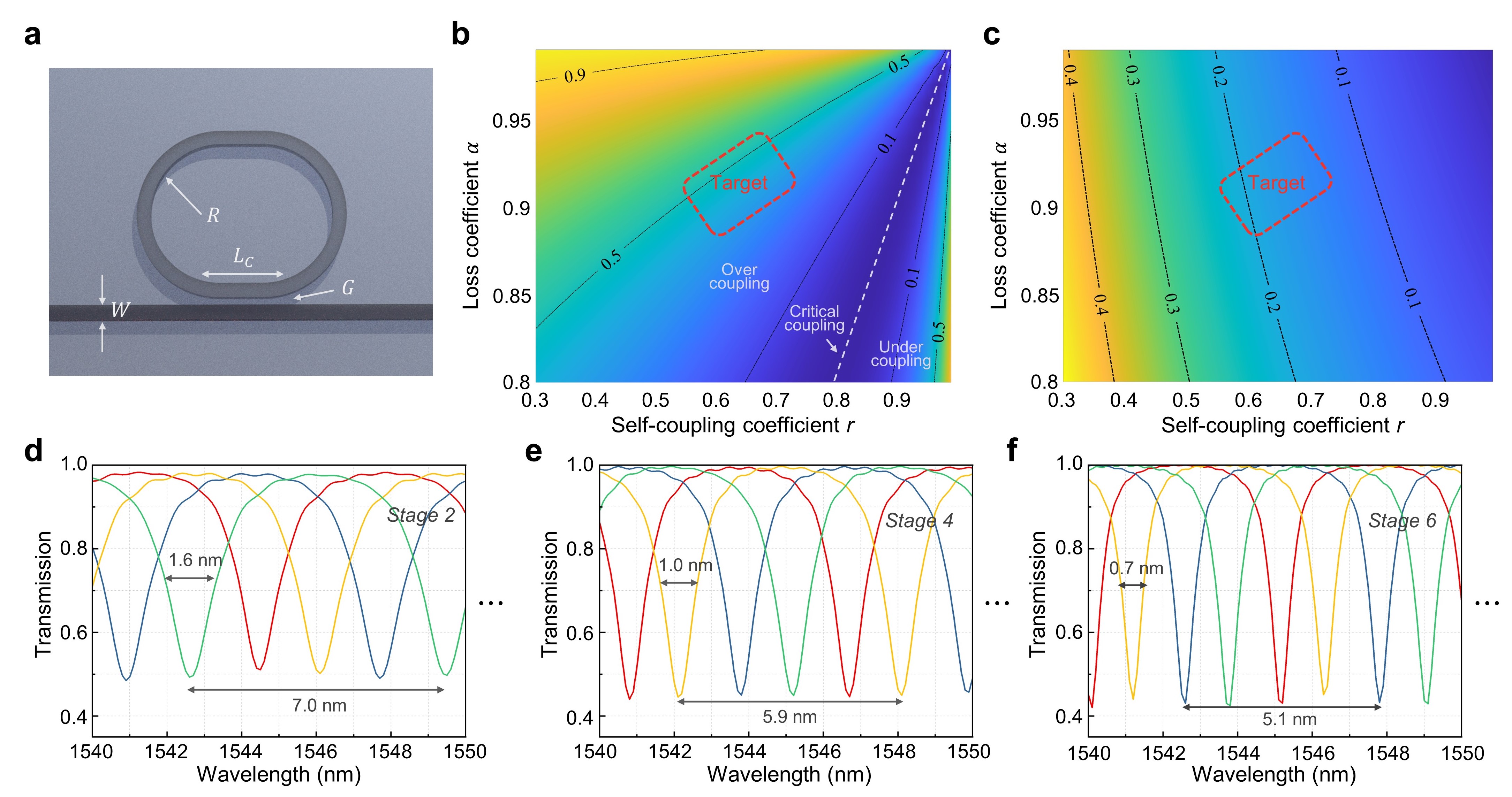


**Figure S2 | Parameter design of the all-pass MRRs.** (a) Schematic of the racetrack MRR. (b-c) The calculated value of $T_{res}$ and the reciprocal of finesse for different combination of $\alpha$ and $r$. The insets show the target range of $\alpha$ and $r$ for the design of distributed spectral filters. (d-f) Simulated transmission spectra of the MRRs at different stages in a 7-stage design. Note that we present the spectrum window between 1540 nm and 1550 nm as an example. Similar periodic pattens are maintained over a broad bandwidth.

**3. Channel diversity**

The channels in meshes of reconfigurable cells may partially overlap, i.e., the channels may share a certain number of same spectral filters. Here, we quantify the diversity between channels by counting the number of distinct MRRs between any configured channel and all other channels. Figure S3 presents the distribution of channels vs. the number of distinct MRRs for the networks with different stage numbers. It can be seen that there are only two channels that pass through one distinct MRR (i.e., the extreme case we discussed in Fig. 2(c)), while most channels have at-least 4 distinct MRRs (even for a 6-stage network). On average, the number of distinct MRRs between channels are 4.48, 5.24, 6.00, and 6.75, for the 6-stage, 7-stage, 8-stage, and 9-stage designs, respectively. These results illustrate that the proposed network topology provides sufficient channel diversity regarding the combination of spectral filters.


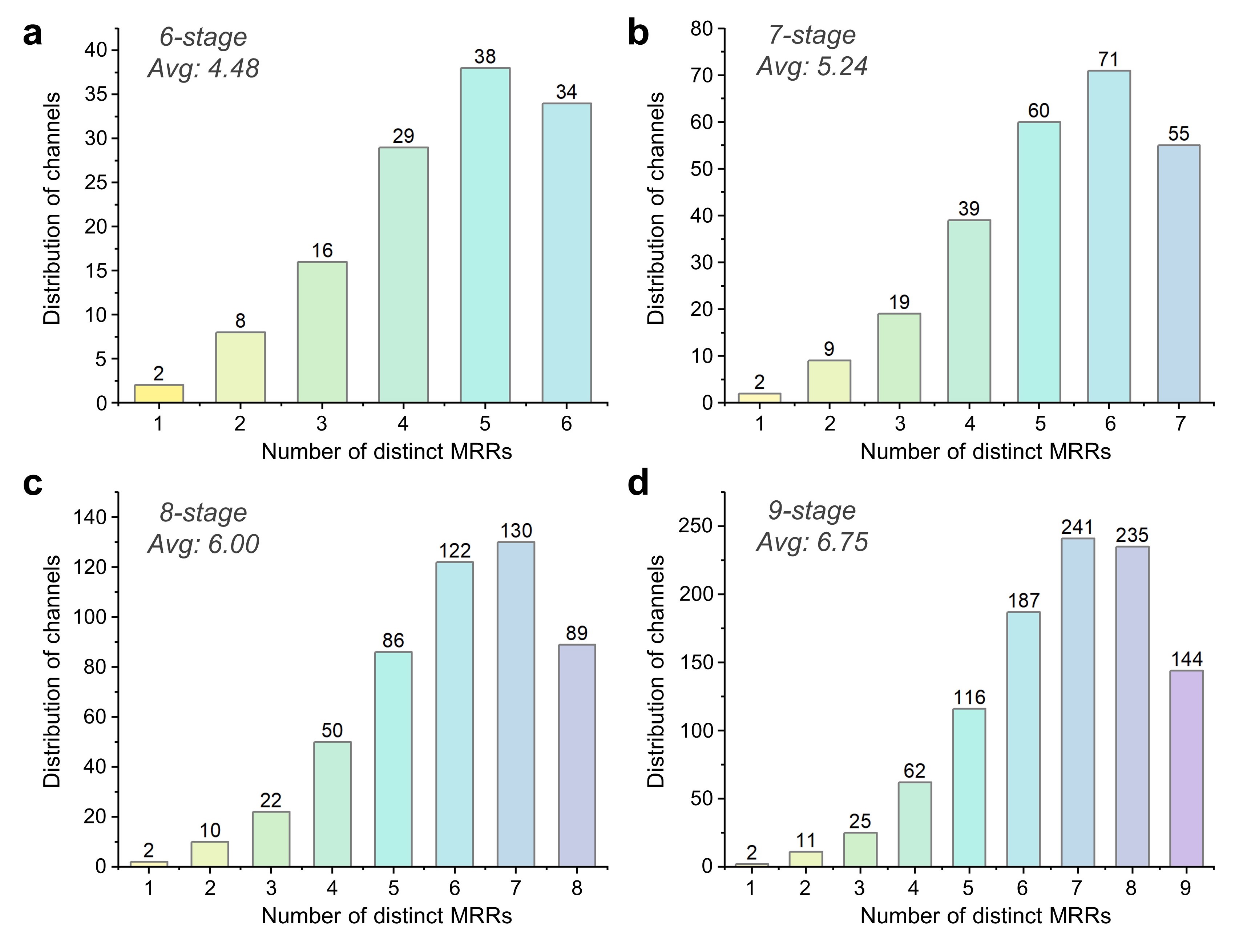


**Figure S3 | Channel diversity regarding the filter combinations.** (a-d) The distribution of channels vs. the number of distinct MRRs for the 6-stage, 7-stage, 8-stage, and 9-stage designs, respectively. The insets show the average number of distinct MRRs between channels.

**4. Experimental set-up**

The experimental set-up for the spectrometer calibration and testing is shown by Fig. S4. To calibrate the channel spectral responses, we launch a broadband ASE source to the chip and use a commercial optical spectral analyzer (YOKAGAWA AQ6370D) to measure the transmission spectra of different channels. After calibration, the input light source is switched to the unknown narrowband or broadband signals under test, while an optical power meter (OPM) is used to measure the output power intensities. An automatic electrical control plane is developed to sweep the bias voltages for the configuration of all sampling channels and collect the real-time data from the photodiode. This system consists of a microcontroller unit (MCU, STM32H7A3) with an embedded analogue-to-digital converter (ADC) module, a multi-channel digital-to-analogue converter (DAC, AD5370), and a customized driving board. The MCU is programmed to transmit the pre-stored voltage in a look-up table to the DAC to generate analog electrical signals. These signals are then amplified by the customized driving board to trigger the reconfiguration of the spectrometer. In the meantime, the output signals from the photodiode are sampled by the ADC on the MCU. In our experiments, this system demonstrates a sampling speed of < 1 ms per channel, such that the total measurement time of 256 channels is within 0.3 seconds. Here, the main bottleneck in speed can be attributed to the limited data transmission rate between the MCU and the DAC. This, however, can be effectively improved by upgrading the microcontroller with more advanced modules or FPGAs and implementing high-speed electrical interconnections.

**5. Thermal stability**


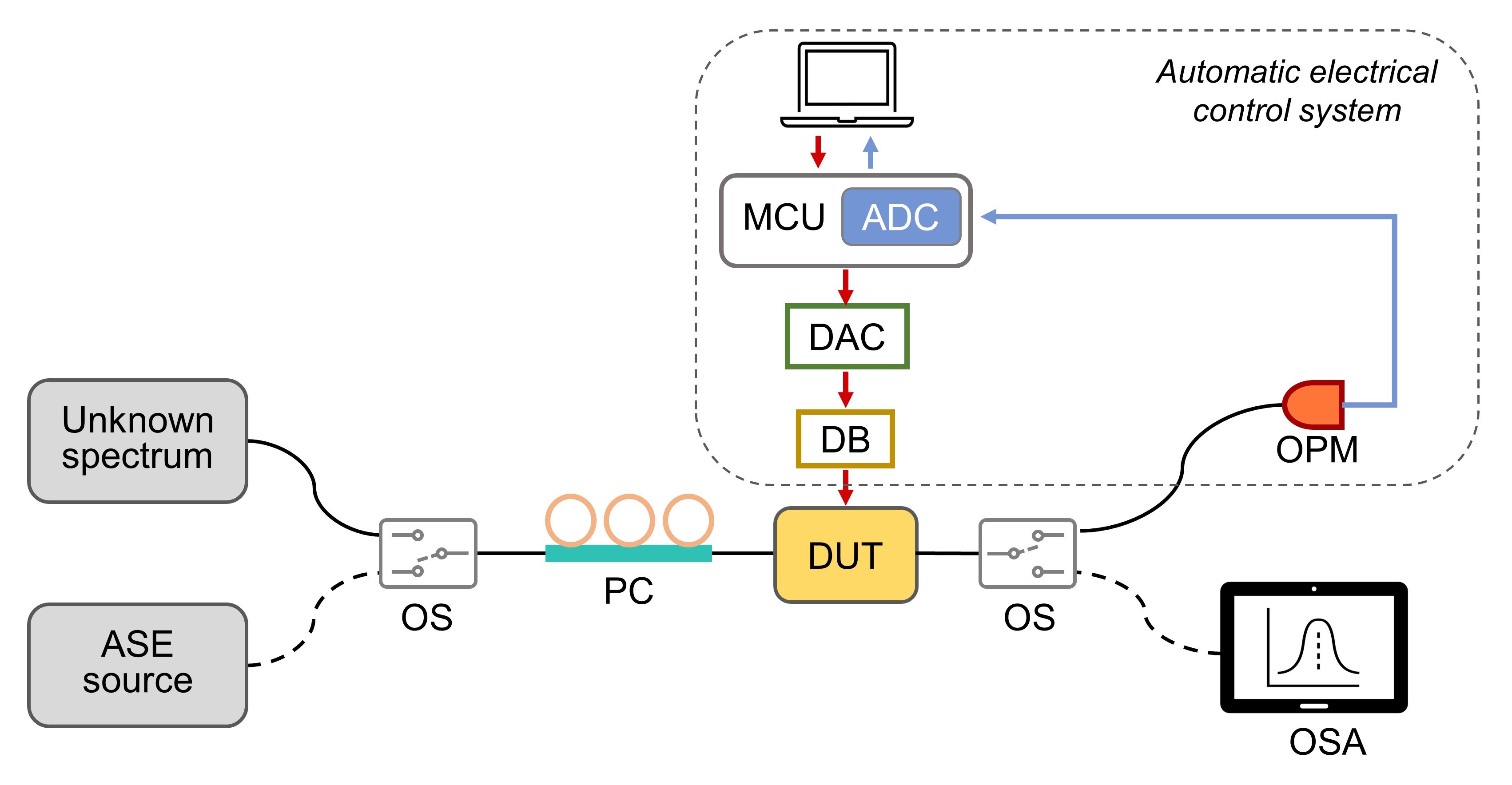


**Figure S4 | Experimental set-up for the spectrometer calibration and testing.** The ASE source and OSA are used for the calibration of channel spectral responses, while the OPM is used for the power measurement of unknown incident spectra. An automatic electrical control system is used to automatically sweep the sampling channels and collect the output data from OPM. OS: optical switch. PC: polarization controller. DUT: device under test. DB: driving board. DAC: digital-to-analogue converter. ADC: analogue-to digital converter. MCU: microcontroller unit. OSA: optical spectrum analyzer. OPM: optical power meter.

To verify the device thermal stability, we measure the channel spectral responses under different temperatures and observe a redshift of 16 pm per degree (see Fig. 5(d)). Accordingly, we simulate the output power at all sampling channels for a narrowband signal with temperature variations, and their normalization to the case with zero temperature change is shown by Fig. S5a. Note that here, the measurement error is also taken into consideration. We then reconstruct the input spectra using the sampling matrix obtained with no temperature change, as shown by Fig. S5b. As can be seen, with up to ± 2.0 °C change of temperature, the input signal can still be recovered to the accuracy of the spectral resolution, i.e., the offset of center wavelength being within ± 30 pm.


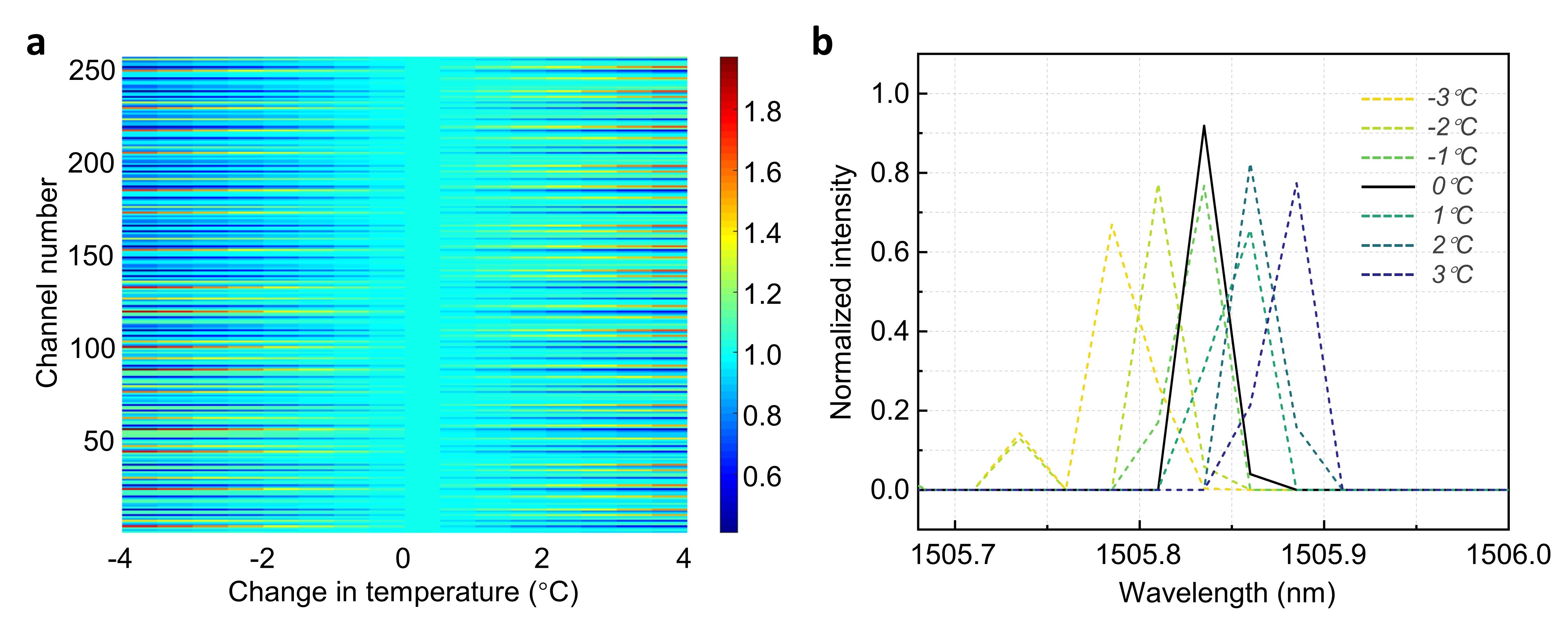


**Figure S5 | Thermal stability of the reconfigurable spectrometer.** (a) Simulated output power at all sampling channels for a narrowband signal with different temperature changes, which is normalized to the case with zero temperature change. (b) Reconstructed spectra at different temperatures, showing a tolerance of ± 2 °C.

**Reference**

S1. Redding, B. Compact spectrometer based on a disordered photonic chip. *Nature Photonics* **7**, 6 (2013).

S2. Yang, Z. *et al.* Single-nanowire spectrometers. *Science* **365**, 1017–1020 (2019).

S3. Bao, J. & Bawendi, M. G. A colloidal quantum dot spectrometer. *Nature* **523**, 67–70 (2015).

S4. Deotare, P. *et al.* On-chip integrated spectrometer using nanobeam photonic crystal cavities. in *Conference on Lasers and Electro-Optics 2012* CM3B.4 (OSA, 2012).

S5. Zhang, Z. *et al.* Compact High Resolution Speckle Spectrometer by Using Linear Coherent Integrated Network on Silicon Nitride Platform at 776 nm. *Laser & Photonics Reviews* 2100039 (2021) doi:10.1002/lpor.202100039.

S6. Yi, D. *et al.* Integrated Multimode Waveguide With Photonic Lantern for Speckle Spectroscopy. *IEEE Journal of Quantum Electronics* **57**, 1–8 (2021).

S7. Hadibrata, W. *et al.* Compact, High‐resolution Inverse‐Designed On‐Chip Spectrometer Based on Tailored Disorder Modes. *Laser & Photonics Reviews* **15**, 2000556 (2021).

S8. Xiong, J. *et al.* One-shot ultraspectral imaging with reconfigurable metasurfaces. *arXiv preprint arXiv:2005.02689* (2020).

S9. Sun, C. *et al.* Scalable On‐Chip Microdisk Resonator Spectrometer. *Laser & Photonics Reviews* 2200792 (2023) doi:10.1002/lpor.202200792.

S10.Xu, H. *et al.* Integrated Single-Resonator Spectrometer beyond the Free-Spectral-Range Limit. *ACS Photonics* acsphotonics.2c01685 (2023) doi:10.1021/acsphotonics.2c01685.

S11.Qiao, Q. *et al.* MEMS-Enabled On-Chip Computational Mid-Infrared Spectrometer Using Silicon Photonics. *ACS Photonics* **9**, 2367–2377 (2022).

S12.Ma, K. *et al.* High-resolution compact on-chip spectrometer based on an echelle grating with densely packed waveguide array. *IEEE Photonics Journal* **11**, 1–7 (2018).

S13.Kyotoku, B. B., Chen, L. & Lipson, M. Sub-nm resolution cavity enhanced micro-spectrometer. *Optics Express* **18**, 102–107 (2010).

S14.Zhu, A. Y. *et al.* Ultra-compact visible chiral spectrometer with meta-lenses. *Apl Photonics* **2**, 036103 (2017).

S15.Zhang, L. *et al.* Ultrahigh-resolution on-chip spectrometer with silicon photonic resonators. *Opto-Electronic Advances* **5**, 210100–210100 (2022).

S16.Zheng, S. *et al.* A Single-Chip Integrated Spectrometer via Tunable Microring Resonator Array. *IEEE Photonics J.* **11**, 1–9 (2019).

S17.Xia, Z. *et al.* High resolution on-chip spectroscopy based on miniaturized microdonut resonators. *Optics Express* **19**, 12356–12364 (2011).

S18.Emadi, A. *et al.* Design and implementation of a sub-nm resolution microspectrometer based on a Linear-Variable Optical Filter. *Optics Express* **20**, 489–507 (2012).

S19.Nezhadbadeh, S. *et al.* Chirped-grating spectrometer-on-a-chip. *Optics Express,* **28**, 24501–24510 (2020).

S20.Souza, M. C. *et al.* Fourier transform spectrometer on silicon with thermo-optic non-linearity and dispersion correction. *Nature communications* **9**, 1–8 (2018).

S21.Zheng, S. N. *et al.* Microring resonator-assisted Fourier transform spectrometer with enhanced resolution and large bandwidth in single chip solution. *Nature communications* **10**, 2349 (2019).

S22.Wang, H. *et al.* On-chip Fourier transform spectrometers by dual-polarized detection. *Optics Letters* **44**, 2923–2926 (2019).

S23.Velasco, A. V. *et al.* High-resolution Fourier-transform spectrometer chip with microphotonic silicon spiral waveguides. *Optics Letters* **38**, 706 (2013).

S24.Kita, D. M. *et al.* High-performance and scalable on-chip digital Fourier transform spectroscopy. *Nature communications* **9**, 4405 (2018).

S25.Paudel, U. & Rose, T. Ultra-high resolution and broadband chip-scale speckle enhanced Fourier-transform spectrometer. *Optics Express* **28**, 16469 (2020).

S26.Halir, R. *et al.* Ultra-broadband nanophotonic beamsplitter using an anisotropic sub-wavelength metamaterial. *Laser & Photonics Reviews* **10**, 1039–1046 (2016).

S27.Tanizawa, K. *et al.* 32× 32 strictly non-blocking Si-wire optical switch on ultra-small die of 11× 25 mm2. *Optical Fiber Communication Conference 2015*. M2B.5. (OFC, 2015).

S28.Bogaerts, W. *et al.* Silicon microring resonators. *Laser & Photonics Reviews* **6**, 47–73 (2012).

S29.Yin, Y.-X. *et al.* High-Q-Factor Silica-Based Racetrack Microring Resonators. *Photonics* **8**, 43 (2021).
